# Microdisplacement Measurement Based on F-P Etalon: Processing Method and Experiments

**DOI:** 10.3390/s21113749

**Published:** 2021-05-28

**Authors:** Xiaoyan Shen, Shinan Zhou, Dongsheng Li

**Affiliations:** College of Metrology and Measurement Engineering, China Jiliang University, Hangzhou 310018, China; p1902085279@cjlu.edu.cn (S.Z.); lidongsheng@cjlu.edu.cn (D.L.)

**Keywords:** microdisplacement measurement, Fabry–Perot etalon, circular regression, multichord averaging method, focusing control

## Abstract

Herein, a processing method is proposed for accurate microdisplacement measurements from a 2D Fabry–Perot (F-P) fringe pattern. The core of the processing algorithm uses the F-P interference imaging concentric ring pattern to accurately calculate the centre coordinates of the concentric ring. The influencing factors of measurement were analysed, and the basic idea of data processing was provided. In particular, the coordinate rotation by the 45-degree method (CR) was improved; consequently, the virtual pixel interval was reduced by half, and the calculation accuracy of the circle centre coordinate was improved. Experiments were conducted to analyse the influence of the subdivision and circle fitting methods. The results show that the proposed secondary coordinate rotation (SCR) by 45 degrees method can obtain higher accuracy of the centre coordinate than the CR method, and that the multichord averaging method (MCAM) is more suitable for calculation of the centre coordinate than the circular regression method (CRM). Displacement measurement experiments were performed. The results show that the standard experimental deviation of the centre of the circle is approximately 0.009 µm, and the extended uncertainty of the displacement measurement in the range of 5 mm is approximately 0.03 μm. The data processing method studied in this study can be widely used in the field of F-P interferometry.

## 1. Introduction

The Fabry–Perot (F-P) etalon is an optical multibeam interference component that can obtain a high-resolution spectrum. After being traditionally used in astronomy since the 1970s [1], it has been widely used in LIDAR instruments for environmental monitoring [2], laser technology [3], atmospheric optics [4,5], marine optics [6], gas analysis [7], length measurement, and angle metrology [8]. The F-P interferometric technology not only includes the F-P etalon but also other structures with F-P interference form such as the optical fibre F-P interferometer [9,10], MEMS F-P interferometer [11,12], and complex mixed F-P interferometric measurement [13,14] of multiple quantities of temperature, displacement, structural loads, electro-optic coefficients, twist/rotation, strain, and pressure [15,16,17,18,19].

The F-P interferometric displacement measurement is particularly attractive because of its intrinsic high resolution compared to the vast majority interferometric methods with two-beam amplitude splitting interferometers. The F-P etalon for displacement measurement has two approaches. In the first approach, the displacement is obtained according to the F-P cavity length, which changes the fringe pattern’s expansion, with nanoscale high resolution but normally small measuring range. (Lawall [20] designed a unique system that measured displacements up to 50 mm, with an uncertainty of 10 pm). In this way, a laser is often used to scan the F-P cavity to measure the displacement dynamically, while the 2D fringe pattern is changed to a 1D signal to simplify and accelerate the process [21].

The fibre extrinsic F-P interferometer (EFPI) is an obvious evolutionary form that is attracting more attention [22,23,24,25]. In the second approach, the displacement is obtained from the movement of the no-expansion fringe pattern with a microscale low-resolution but normally large measuring range. However, its simple measurement principle has inspired little research. There is no denying that it is an effective, easy-to-use, and multidirectional measurement. For this simple method, the resolution of the displacement is pivotally determined by the resolution of the 2D fringe pattern.

In fact, the resolution of the fringe pattern is a general and common problem in all F-P etalon measuring systems. Therefore, the pixel subdivision algorithm is commonly employed in the process for all image measurement requirements. For the demodulation of star sensor images, Wang et al. [26] proposed a new Gaussian centroid extracting subpixel positioning method, which has higher precision than those of traditional grey-weighted centroid methods. Yang [27] proposed a new subpixel subdivision location algorithm for estimating the position of the star on a focal plane array, combining a weighted scheme for centroiding. The simulation results showed that the accuracy of the star position can reach 1/150 pixels. For the geometry size measurement from the image, Binuse [28] improved binomial interpolation to improve the subpixels for edge fine positioning in noncontact measurement of the critical part size. For displacement and angle measurement, Yu [29] proposed a robust subpixel subdivision algorithm based on the least squares method, which is more accurate and has better robustness than the traditional algorithm (centroid algorithm). In particular, patented CCD pixel subdivision algorithms are used for collimators [30,31]. A smooth filter is also used to reduce the signal noise and to promote the measurement accuracy. As mentioned in the literature [2], the wavelet denoising filter, mean filter, and FFT low-pass filter are three popular smoothing filters, each of which has application characteristics.

This paper focuses on the second displacement measurement method based on the basic F-P etalon’s interference imaging optical system, including the processing method and experiments. For the processing method, we proposed a novel 2D pixel subdivision method using a large number of pixels, which is based on the secondary coordinate rotation (SCR) by 45 degrees method, divides the unit from one pixel into smaller units, and has a similar mean smoothing effect as well. Then, we chose the best ring pattern to evaluate the centre of the concentric rings by using the multichord average method (MCAM) compared with the circular regression method (CRM). More important, the accuracy can be calculated theoretically using mathematical statistics from the primary experiments. This processing method can be extended to other measurement applications using F-P and area-array detectors.

## 2. Materials and Methods

### 2.1. Optical Sketch

**Figure 1:**
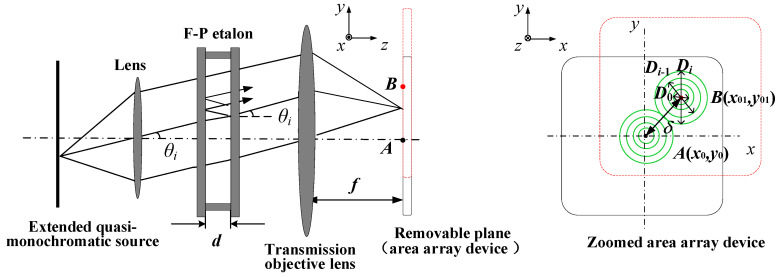
Interference imaging principle of F-P etalon.

A schematic of the experimental setup is presented in Figure 1. After passing through the lens, the quasi-monochromatic light is coupled into the F-P etalon with an air-gap spacing *d*, and a series of conical beams are formed on the exit surface. After the conical beams pass the objective lens, concentric interference rings are generated on the focal plane; these can be recorded by an area-array detector as 2D pattern information. According to the principles of optics, a simplified equation for F-P interference imaging is [32,33]:

(1) $${\left(\frac{D_{i}/2}{f}\right)}^{2}={\left(\tan\theta_{i}\right)}^{2}={\left(\frac{k_{0}+\varepsilon }{k_{0}-i}\right)}^{2}-1$$

where *i* is the ring order of the concentric interference rings from the centre, $i=0,1,2,\ldots ,i_{\max}$, $D_{i}$ is the diameter of the $i^{\mathrm{th}}$ ring, *f* is the focal length of the imaging lens, $\theta_{i}$ is the incident angle of the conical beam causing the $i^{\mathrm{th}}$ ring, $k_{0}$ and ε are the integer and fractional parts of the interference order of the ring in the centre, respectively, where 0≤ε<1. Further, $k_{0}+\varepsilon =2dn/\lambda_{0}$ ( $\lambda_{0}$ is the vacuum wavelength of the extended quasi-monochromatic light source, d is the air-gap spacing of the F-P etalon, *n* is the air refractive index in the gap), $k_{0}$ is the interference order of the $0^{\mathrm{th}}$ ring, and $\left(k_{0}-i\right)$ is the $i^{\mathrm{th}}$ ring’s interference order. In Equation (1) $\theta_{i}$ is related to $D_{i}$ and corresponds to the ring order value *i* or the interference order $\left(k_{0}-i\right)$ of the $i^{\mathrm{th}}$ ring. Therefore, we can preliminarily obtain $D_{i}$ under different *i* when the parameters ( $\lambda_{0}$, *d*, *n*, and *f*) are determined. Although $D_{i}$ is estimated, it is efficient in analysing the imaging characteristics and guiding the experiments. Although Equation (1) is significantly important, it is not directly implemented for displacement measurements.

The formula directly adopted for the displacement measurement is expressed in Equation (2). As illustrated in Figure 1, when the removable plane shifts, the F-P imaging pattern moves in the focal plane. Theoretically, any dot in the pattern can reflect the movement change, however, a change in the centre dot will yield the best results. Therefore, the displacement δ should be drawn from the two centre dots *A* $\left(x_{0},y_{0}\right)$ and *B* $\left(x_{01},y_{01}\right)$:

(2) $$\delta =\sqrt{\delta_{x}{}^{2}+\delta_{y}{}^{2}}=\sqrt{{\left(x_{01}-x_{0}\right)}^{2}+{\left(y_{01}-y_{0}\right)}^{2}}$$

Clearly, the displacement δ can be accurately measured based on the accurate acquisition of coordinates $\left(x_{0},y_{0}\right)$ and $\left(x_{01},y_{01}\right)$. Thus, the measurement process is very simple, with only three steps. The first step is to choose the light source of $\lambda_{0}$ the lens, F-P etalon *d*, objective lens *f*, and a suitable area-array detector (located in the focal plane of the objective lens). The second step is to obtain the fringe pattern on the detector and calculate its centre coordinates $\left(x_{0},y_{0}\right)$. The third step is to obtain the fringe pattern after moving the plane and then calculate $\left(x_{01},y_{01}\right)$ to finally obtain δ according to Equation (2).

### 2.2. Analysis of Influencing Factors

**Figure 2:**
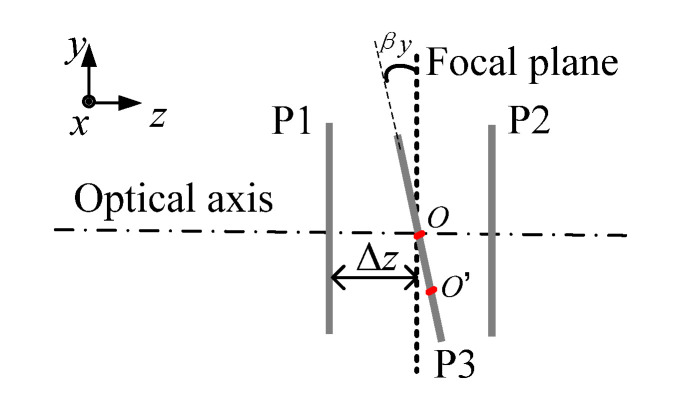
Area-array device deviating from focal plane.

Apparently, the accuracy of the displacement δ was mainly affected by the accuracy of the centre coordinates. The influencing factors of the error involved in the practical measurement process are as follows:

- Error of the pixel interval of the area-array detector, $\Delta_{w}$. It includes the temperature-induced rectifiable systematic error and manufacturing-induced random error. The average pixel interval w is used as the unit to express displacement as much as possible, and the experiment was set up in a temperature-controlled lab to reduce $\Delta_{w}$.
- Random error of the photoelectric signal conversion rate $\Delta_{R}$. This is mainly owing to the manufacturing-induced random error and the experimental setup, causing random errors in the photoelectric image signal. This error component can be effectively reduced by the method of virtual pixel subdivision and smoothing (described in Section 2.3.2).
- The influence of the area-array detector deviating from the focal plane, which is mainly caused by the *z*-direction displacement $\Delta_{z}$, *x*-axis deflection $\beta_{x}$, and *y*-axis deflection $\beta_{y}$. As shown in Figure 2, $\Delta_{z}$ exists when the area-array detector is parallel but not in the focal plane, located at positions P1 or P2. In this case, off-focus imaging occurs, which affects the imaging quality and blurs the interference pattern. If there is $\beta_{y}$ when the area-array detector rotates to P3, then the interference pattern exhibits an ellipse phenomenon. When the centre moves from *O* to *O*’, off-focus imaging occurs as well, and the pattern is further blurred. If there is $\beta_{x}$, then the same situation will occur as in the situation with $\beta_{y}$. When $\Delta_{z}$, $\beta_{x}$, and $\beta_{y}$ exist simultaneously, the situation becomes more complicated. For practical measurement, we adjusted the position of the area-array detector through multiple adjustments and attempted to find the optimal focal plane position according to Section 3.2.
- Influence of massive pixel data information selection in the area-array detector. Not all of the information of the 2D pattern needs to be applied to calculate the centre coordinates because unfavourable ring data will increase the calculation difficulty or affect the calculation accuracy. Therefore, it is necessary to extract effective pixel data. In this study, we analysed a large number of ring data and obtained the optimal ring.
- Accuracy of ring peak coordinates. The ring peak coordinates (coordinates of the maximum of photoelectric intensity on each interference ring) are required regardless of the specific method used to obtain the centre coordinates. Instead of directly using the pixel coordinates, the coordinates of the ring peaks can be calculated accurately by the local univariate quadratic regression method, as well as by reducing the algorithm error in the centre solving process, so that the accuracy of the displacement measurement can be improved (see Section 2.3.1) for the detailed calculation process.
- Influence of the calculation method of the ring centre. The measurement error is w/2 by directly extracting the pixel coordinates, which is far from the demand. Therefore, the pixel subdivision method was used, as described in Section 2.3.2, and was conducted in Section 3.3. Specific calculation methods were employed to obtain the centre coordinates accurately, such as the classic CRM and the MCAM. Their theories are explained in Section 2.3.3, and the experiments are presented in Section 3.4.

### 2.3. Processing Method on 2D Pattern

**Figure 3:**
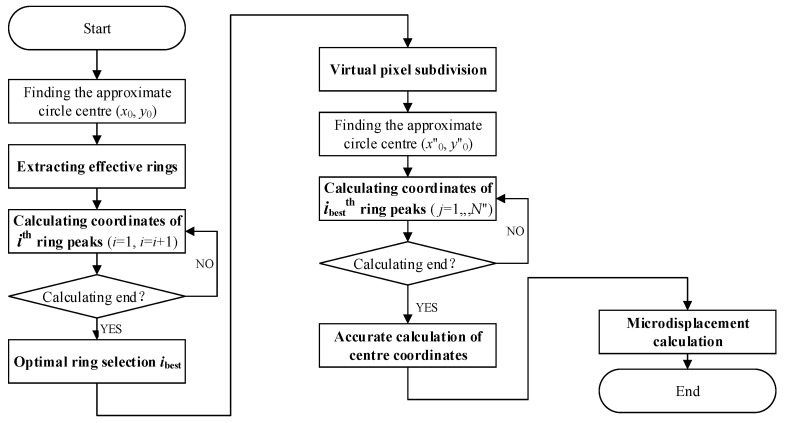
The flow chart of the data processing process.

This section discusses the data processing method mentioned in this paper. In our previous study [33], a virtual pixel subdivision method based on the CR method was proposed to measure displacement, whose data processing process contains extracting effective rings, calculating coordinates of ring peaks, optimal ring selection, virtual pixel subdivision, accurate calculation of centre coordinates, and microdisplacement calculation, as shown in the Figure 3. Among them, ‘calculating coordinates of ring peaks’ is used for the next steps of ‘optimal ring selection’ and ‘accurate calculation of centre coordinate’, as a very basic work that is introduced here first in Section 2.3.1. Then, the improved SCR by the 45-degree method is depicted, as well as the CR method in Section 2.3.2. In addition, the two principles of calculating methods of the MCAM and the CRM are discussed in Section 2.3.3.

#### 2.3.1. Calculating Coordinates of Ring Peaks

**Figure 4:**
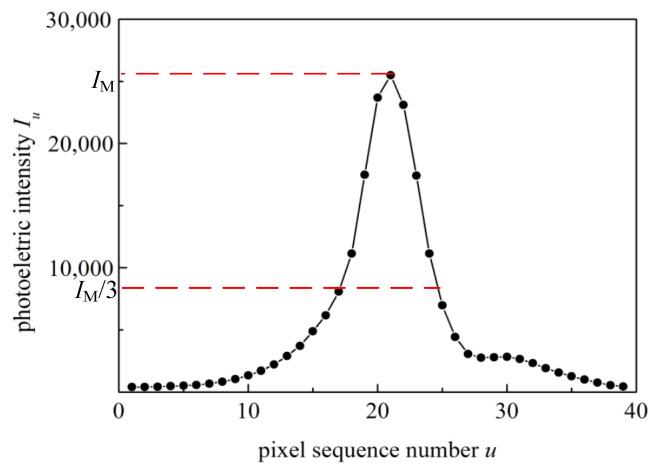
The photoelectric intensity distribution curve.

The coordinates of the ring peaks mainly use the local univariate quadratic regression method. Its basic idea is as follows: (1) Extract the approximate diameter $y=y_{P}$ ($y_{P}$ is the pixel sequence number in the *y*-direction nearly close to the centre, an integer) parallel to the *x*-axis, then intersect it with an interference ring to obtain *m* continuous pixels in the *x*-direction, noting the photoelectric intensity of *m* pixels as $I_{u}$ (*u* is the pixel sequence number in the *x*-direction, an integer). The typical photoelectric intensity distribution of the continuous pixels is shown in Figure 4. (2) Data interception under a threshold, that is, take part of *m* pixels whose photoelectric intensity $I_{u}$>$I_{M}/3$, [$I_{M}=\max\left(I_{u}\right)$]. (3) Set the function of $Z_{u}=I_{M}/I_{u}-1=\alpha_{0}+\alpha_{1}u+\alpha_{2}u^{2}$, and then obtain the regression coefficients of $\alpha_{0}$, $\alpha_{1}$, and $\alpha_{2}$. The coordinates of the ring peak are calculated from the extreme point coordinate $x_{p}=-\alpha_{1}/2\alpha_{2}$ ($x_{p}$ is a non-integer), and the standard deviation $s_{x_{p}}$ can also be calculated. (4) Steps (1)–(3) are used to extract the approximate diameter $x=x_{{}_{P}}$ parallel to the *y*-axis and calculate the ring peak coordinate $y_{p}$ and its standard deviation $s_{y_{p}}$. (5) Finally, the ring peak coordinates $\left(x_{p},y_{p}\right)$ can be obtained precisely using the coordinates’ standard deviations $s_{x_{p}}$ and $s_{y_{p}}$.

#### 2.3.2. Virtual Pixel Subdivision and Smoothing in SCR

**Figure 5:**
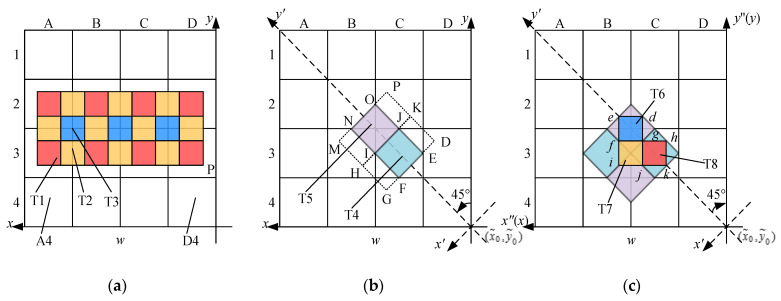
Virtual pixel subdivision methods: (**a**) the average interpolation method; (**b**) the CR method [33]; and (**c**) the SCR method.

The SCR method is proposed and analysed with the previously proposed CR method. Their principles are described here, and the experimental results are presented in Section 3.3. The area-array detector collects F-P interference concentric ring information, and the pixel subdivision is an important way to improve the measurement accuracy. Interpolation is a common method used for pixel subdivisions. As shown in Figure 5a, two-dimensional array pixels can obtain subpixels with an interval of w/2 after average interpolation. There are three different types of subpixels: T1, T2, and T3. T1 is only located in and related to pixel A3, T2 is affected by two adjacent pixels A3 and B3, and T3 is affected by four adjacent pixels (A2, A3, B2, B3). Therefore, the smoothing effects of the different types of subpixels obtained after interpolation are inconsistent. Reference [33] provided a CR method for pixel subdivision and smoothing so that each subpixel is affected by at least four pixels, as shown in Figure 5b. With this method, while improving the smoothing effect of the subpixels, the influence of random error caused by geometric centre deviation and photoelectric conversion rate is reduced, with a subpixel interval of $w\prime =w/\sqrt{2}$. In this study, this method was improved to the SCR method, as shown in Figure 5c.

The SCR method is a subdivision method based on the CR method. First, we find the pixel $\left({\tilde{x}}_{0},{\tilde{y}}_{0}\right)$ closest to the ring centre. ‘Calculating coordinates of ring peaks’ (Section 2.3.1) is employed to obtain two intersection points between a certain chord (*x*-direction or *y*-direction) and a certain ring. The middle point of the two intersection points is used to determine ${\tilde{x}}_{0}$ or ${\tilde{y}}_{0}$; ${\tilde{x}}_{0}$ and ${\tilde{y}}_{0}$ are the pixel sequence numbers in the *x*- and *y*-directions, respectively, and they are integers. Second, as shown in Figure 5b, the *x*-axis and *y*-axis are rotated by 45 degrees with $\left({\tilde{x}}_{0},{\tilde{y}}_{0}\right)$ as the ring centre to obtain a new *x*′-axis and *y*′-axis. Third, ‘virtual pixel subdivision and smoothing’ were performed to obtain two different types of subpixels. These are subpixels T4 and T5, respectively. T4 is the small square FIJE inscribed in the centre of pixel C3. T5 is a small square INOJ composed of original pixels B2, B3, C2, and C3. The average photoelectric intensity of rectangular GHKD and rectangular HMPK is taken as the photoelectric intensity of T4 and T5, respectively. Thus, Equations (3)–(5) are deduced as [33]:

(3) $$w_{\mathrm{INOJ}}^{\prime }=w_{\mathrm{FIJE}}^{\prime }=w/\sqrt{2}$$

(4) $$\begin{array}{ll} Z_{\mathrm{FIJE}} & =\frac{1}{2}\left(\frac{3}{4}Z_{C3}+\frac{1}{16}Z_{B3}+\frac{1}{16}Z_{C2}+\frac{1}{16}Z_{C4}+\frac{1}{16}Z_{D3}\right)\\ & =\frac{3}{8}Z_{C3}+\frac{1}{32}\left(Z_{B3}+Z_{C2}+Z_{C4}+Z_{D3}\right) \end{array}$$

(5) $$Z_{\mathrm{INOJ}}=\frac{3}{16}\left(Z_{B3}+Z_{C2}\right)+\frac{1}{16}\left(Z_{B2}+Z_{C3}\right)$$

where ${w^{\prime }}_{}$ is the subpixel interval, and *Z* is the photoelectric intensity. Fourth, as shown in Figure 5c, the *x*′-axis and *y*′-axis are rotated by 45 degrees in reverse to construct the *x*″-axis and *y*″-axis, and then the subpixels are constructed as described in the third step to obtain three types of subpixels T6, T7, and T8. The acquisition of their photoelectric intensity is the same in the third step, which is to construct a rectangle and take the average photoelectric intensity of the rectangle as the photoelectric intensity of the subpixel. For different types of subpixels, the interval $w^{\prime \prime }$ and the photoelectric intensity after secondary construction are

(6) $$w^{\prime \prime }=w/2$$

(7) $$\begin{array}{ll} Z_{defg} & =\frac{43}{256}\left(Z_{B3}+Z_{C2}\right)+\frac{19}{256}\left(Z_{B2}+Z_{C3}\right)\\ & +\frac{1}{512}\left(Z_{A2}+Z_{A3}+Z_{B1}+Z_{B4}+Z_{C1}+Z_{C4}+Z_{D2}+Z_{D3}\right) \end{array}$$

(8) $$\begin{array}{ll} Z_{gfij} & =\frac{47}{256}\left(Z_{B3}+Z_{C3}\right)+\frac{9}{256}\left(Z_{B4}+Z_{C2}\right)\\ & +\frac{5}{256}\left(Z_{B2}+Z_{C4}\right)+\frac{3}{256}\left(Z_{A3}+Z_{D3}\right) \end{array}$$

(9) $$\begin{array}{ll} Z_{hgjk} & =\frac{5}{16}Z_{C3}+\frac{5}{128}\left(Z_{B3}+Z_{C2}+Z_{C4}+Z_{D3}\right)\\ & +\frac{3}{256}\left(Z_{B4}+Z_{D2}\right)+\frac{1}{256}\left(Z_{B2}+Z_{D4}\right) \end{array}$$

**Figure 6:**
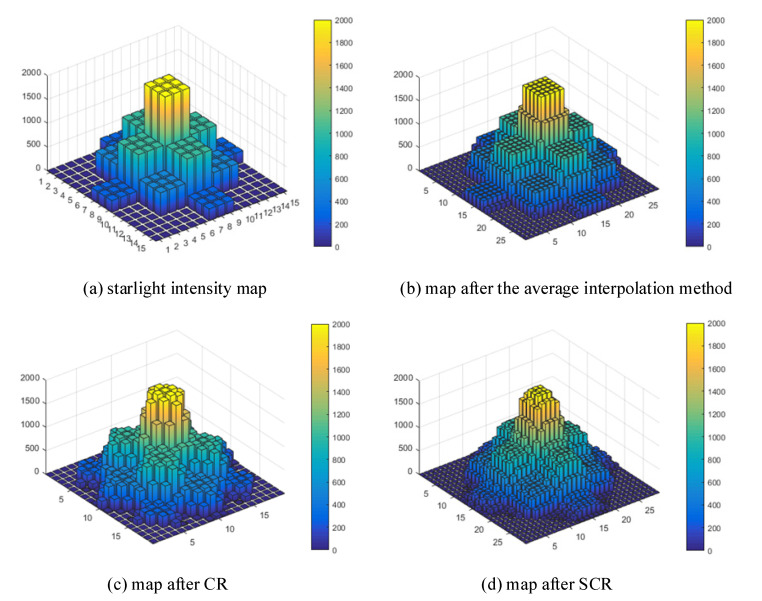
Pixel subdivision results by different interpolation methods.

Figure 6 shows the pixel subdivision results obtained by the average interpolation method, the CR method, and the SCR method of the two-dimensional typical star point light intensity map. It can be clearly seen from Figure 6 that the two-dimensional star points light intensity after SCR method has been significantly subdivided and smoothed.

#### 2.3.3. Accurate Calculation of the Centre Coordinates

**Figure 7:**
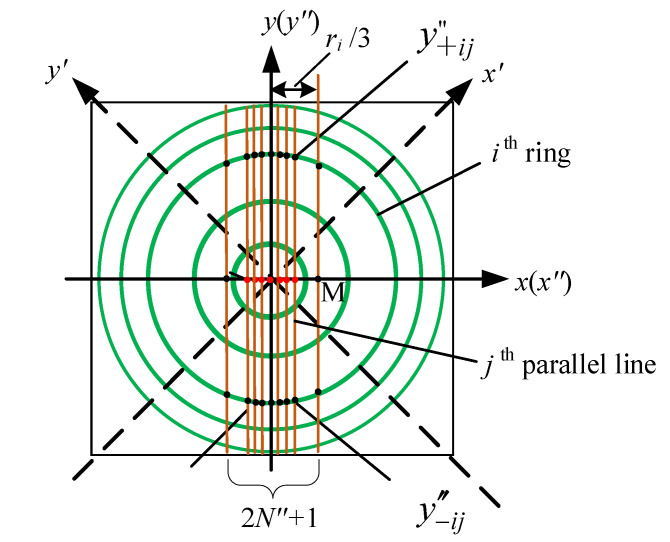
Multiple ring peak coordinates under line segments *N*″.

(1) Line segments *N*″ to obtain multiple ring peak coordinates: Whether using CRM or MCAM to calculate accurate centre coordinates, we need to obtain multiple available coordinates of ring peaks $\left(x_{p}^{\prime \prime },y_{p}^{\prime \prime }\right)$ on the basis of ‘calculating coordinates of ring peaks’, which contains two issues. The first is which ring or rings to choose. In fact, we can quickly determine the ring order of the optimal ring by calculating the ring peak coordinates on different rings and their standard deviations, as pointed out in our experimental research. The second is how to determine the optimal amount of available ring peak coordinates on the optimal ring. There is an important parameter *N*″ denoting the number of straight lines on one side, as shown in Figure 7. Owing to the need for a fitting effect, the intersection of the *N*″th straight line and the *x*-axis does not exceed one-third the radius of the ring, shown as point M in Figure 7. As shown in Figure 7, after finding the approximate centre point in the subdivided pattern and then establishing parallel lines on both sides parallel to the *x*″-axis (or *y*″-axis), a total of $2\times \left(2N^{\prime \prime }+1\right)=4N^{\prime \prime }+2$ parallel lines in the *x*″-direction and *y*″-direction are determined. These parallel lines intersect with the optimal ring to obtain $2\times 2\times \left(2N^{\prime \prime }+1\right)=8N^{\prime \prime }+4$ coordinates of $x_{p}^{\prime \prime }$ and $y_{p}^{\prime \prime }$, which can be used to calculate the centre coordinates. Here, we explain the subdivided interference patterns after SCR method. Of course, the same processing method is used for the interference pattern without subdivision, and *N*″ is best expressed as *N*.

(2) Calculating the ring centre using MCAM [33]: This method is relatively simple. Using the aforementioned (8*N*″ + 4) ring peak coordinates of $x_{p}^{\prime \prime }$ and $y_{p}^{\prime \prime }$, $x_{0ij}^{\prime \prime }$ and $y_{0ij}^{\prime \prime }$ (*j* = 0, 1, 2, …*N*″) can be obtained, and then the average values can be used as centre coordinates $\left(x_{0i}^{\prime \prime },y_{0i}^{\prime \prime }\right)$ of each ring.

Taking the $y^{\prime \prime }$-direction as an example, as shown in Figure 7, the *j*th line parallel to the $y^{\prime \prime }$-axis intersects the *i*th ring to obtain the coordinate of ring peaks $y_{+ij}^{\prime \prime }$ and its standard deviation $S_{y_{+ij}^{\prime \prime }}$, and the coordinate of ring peaks $y_{-ij}^{\prime \prime }$ and its standard deviation $S_{y_{-ij}^{\prime \prime }}$, so we can obtain the ring centre ordinate $y_{0ij}^{\prime \prime }$ and its standard deviation $S_{y_{0ij}^{\prime \prime }}$:

(10) $$y_{0ij}^{\prime \prime }=(y_{+ij}^{\prime \prime }+y_{-ij}^{\prime \prime })/2$$

(11) $$S_{y_{0ij}^{\prime \prime }}=\sqrt{{\left(S_{y_{+ij}^{\prime \prime }}\right)}^{2}+{\left(S_{y_{-ij}^{\prime \prime }}\right)}^{2}}/2$$

Obviously, there are (2*N*″ + 1) lines that intersect with the *i*th ring, so there will be (2*N*″ + 1) of $y_{0ij}^{\prime \prime }$. The equal-weighted average $\bar{y_{0i}^{\prime \prime }}$ and its standard deviation $S_{\bar{y_{0i}^{\prime \prime }}}$ are as follows:

(12) $$\bar{y_{0i}^{\prime \prime }}=\sum_{j=1}^{2N^{\prime \prime }+1}y_{0ij}^{\prime \prime }/(2N^{\prime \prime }+1)$$

(13) $$S_{\bar{y_{0i}^{\prime \prime }}}=\sqrt{\sum_{j}{\left(y_{0ij}^{\prime \prime }-\bar{y_{0i}^{\prime \prime }}\right)}^{2}/\left(2N^{\prime \prime }\left(2N^{\prime \prime }+1\right)\right)}$$

Similarly, the equal-weighted average $\bar{x_{0i}^{\prime \prime }}$ and its standard deviation $S_{\bar{x_{0i}^{\prime \prime }}}$ of the *i*th ring along the $x^{\prime \prime }$-direction are

(14) $$\bar{x_{0i}^{\prime \prime }}=\sum_{j=1}^{2N^{\prime \prime }+1}x_{0ij}^{\prime \prime }/\left(2N^{\prime \prime }+1\right)$$

(15) $$S_{\bar{x_{0i}^{\prime \prime }}}=\sqrt{\sum_{j}{\left(x_{0ij}^{\prime \prime }-\bar{x_{0i}^{\prime \prime }}\right)}^{2}/\left(2N^{\prime \prime }\left(2N^{\prime \prime }+1\right)\right)}$$

Therefore, from Equations (10)–(15), the centre coordinates $\left(x_{0i}^{\prime \prime },y_{0i}^{\prime \prime }\right)$ (i.e., $\left(\bar{x_{0i}^{\prime \prime }},\bar{y_{0i}^{\prime \prime }}\right)$) under the MCAM for the *i*th ring and their standard deviations $S_{x_{0i}^{\prime \prime }}$ and $S_{y_{0i}^{\prime \prime }}$ (which are $S_{\bar{x_{0i}^{\prime \prime }}}$ and $S_{\bar{y_{0i}^{\prime \prime }}}$) are obtained. Of course, the *i*th ring should be the optimal $i_{\mathrm{best}}$ ring we chose. Furthermore, the corresponding ring radius *r_ij_* can be obtained based on the geometric relationship. Taking the *y*″-direction as an example, the ring radius $r_{yij}^{\prime \prime }$ under the *i*th ring and *j*th line is

(16) $$r_{yij}^{\prime \prime }=\sqrt{{\left[\left(y_{+ij}^{\prime \prime }-y_{-ij}^{\prime \prime }\right)/2\right]}^{2}+d_{j}{}^{2}}$$

where *d_j_* represents the distance from the *j*th line to the centre of the ring $\left(x_{0i}^{\prime \prime },y_{0i}^{\prime \prime }\right)$. Identically, the ring radius *r_xij_* in the *x*″ direction can be obtained. In all, a total of $2\times \left(2N^{\prime \prime }+1\right)$ radii can be obtained in the two directions. Thus, the *i*th ring’s radius *r_i_* can be obtained:

(17) $$r_{i}=\bar{r_{i}}=\left(\sum_{j=1}^{2N^{\prime \prime }+1}r_{yij}+\sum_{j=1}^{2N^{\prime \prime }+1}r_{xij}\right)/\left(4N^{\prime \prime }+2\right)$$

(3) Calculating ring centre using CRM: According to the classic circular regression method [34], using $\left(8N^{\prime \prime }+4\right)$ ring peak coordinates, the estimated ring radius $r_{i}$ and centre coordinates $\left(x_{0i}^{\prime \prime },y_{0i}^{\prime \prime }\right)$ can be obtained by LSM. To complete the calculation of the ring centre and its standard deviation, the calculation process is divided into the following steps.

(1) Take the distance $r_{ip}=\sqrt{{\left(x_{p}^{\prime \prime }-u_{0i}^{\prime \prime }\right)}^{2}+{\left(y_{p}^{\prime \prime }-v_{0i}^{\prime \prime }\right)}^{2}}$ between the $\left(8N^{\prime \prime }+4\right)$ ring peak coordinates and the approximate centre coordinates $\left(u_{0i}^{\prime \prime },v_{0i}^{\prime \prime }\right)$ as the dependent variable, and take the intermediate variable $\cos\theta_{i}=(x_{p}^{\prime \prime }-u_{0i}^{\prime \prime })/r_{ip}$ and $\sin\theta_{i}=(y_{p}^{\prime \prime }-v_{0i}^{\prime \prime })/r_{ip}$ as two independent variables.
(2) Use the model $r_{ip}=r_{i}+x_{0i}^{\prime \prime }\cos\theta_{i}+y_{0i}^{\prime \prime }\sin\theta_{i}$ to make a bivariate LSM regression to obtain the coefficients $r_{i}$, $x_{0i}^{\prime \prime }$, $y_{0i}^{\prime \prime }$ and their standard deviations $s_{r_{i}}$, $s_{x_{0i}^{\prime \prime }}$, $s_{y_{0i}^{\prime \prime }}$.
(3) Take the coordinates obtained by regression as $\left(u_{0i}^{\prime \prime },v_{0i}^{\prime \prime }\right)$ and repeat the operations of (1) and (2) to further reduce the standard deviations $s_{r_{i}}$, $s_{x_{0i}^{\prime \prime }}$, $s_{y_{0i}^{\prime \prime }}$, until the iteration error is less than $1\times 10^{-5}w^{\prime \prime }$.

## 3. Experiments and Results

### 3.1. Experimental Setup

**Figure 8:**
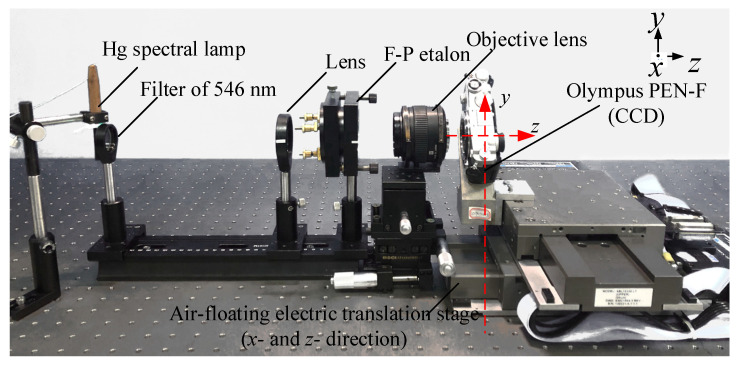
Experimental setup for microdisplacement.

**Figure 9:**
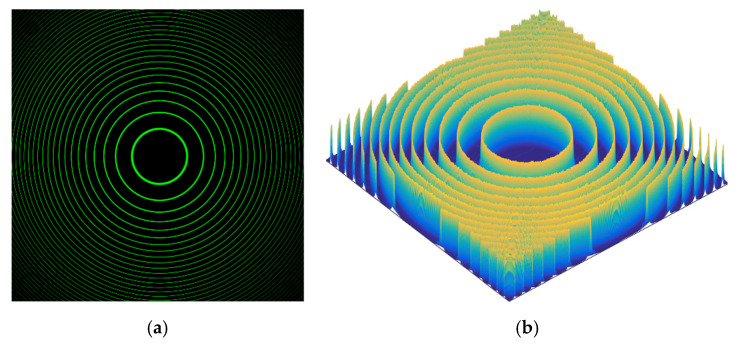
Concentric interference ring image from experimental setup: (**a**) high-brightness concentric interference ring image and (**b**) the 3D image of the light intensity signal of the concentric interference ring.

The experimental device shown in Figure 8 [35], a Hg spectral lamp was the light source, the filter’s wavelength was λ=546 nm, and the F-P etalon’s air-gap spacing *d* was approximately 2 mm. The OLYMPUS-produced objective lens has a fixed focal lens of *f* = 50 mm, and an Olympus PEN-F camera was used to collect the concentric interference ring pattern. The area-array size of the CCD was 17.4 × 13.0 mm, the number of pixels was 5184 × 3888, and the average pixel size was w≈3.35 μm. After adjusting all parts to be coaxial, the light entered the F-P etalon evenly. By adjusting the CCD to be in the focal plane, the interference image was obtained. The movements in the *x*-direction for displacement and *z*-direction for focusing were provided by an AEROTECH ABL100 2D air-flotation electric translation stage with a repetitive positioning accuracy of approximately 50 nm. The F-P interference concentric ring pattern obtained from the experiments is shown in Figure 9a with a picture size of approximately 115 M.

### 3.2. Optimal Ring and Focusing

When the CCD is adjusted to the focal plane, the *i*th ring (*i* = 7, 8, 9, …, 16) coordinates of ring peaks from the image shown in Figure 9 (see Section 2.3.1) and the optimal ring of the 9th ring are determined, that is, *i*_best_ = 9. Note that the focusing position is not the optimal focal plane position but is irrelevant to the best ring. When focusing precisely, we need to control the AEROTECH stage to move along the +*z*-direction, in detail moving from a location of near defocus (setting the relative focusing distance at 0 μm) in 10-μm steps to a far defocus location with a set of ‘defocus-focus-defocus’ adjustments. In this process, we collected a total of 18 image positions.

Therefore, we calculated the full width at half maximum (FWHM) of the 9th ring in 18 patterns. The FWHM can calculate the fineness of the F-P interference fringes and judge the image quality of the fringes. From the results shown in Table 1, it is shown that the measured minimum value of FWHM is 3.949 w (here, w is the average pixel interval, to be seen as the unit), located near a relative focusing distance of 80 μm. Therefore, we obtained the optimal focal plane position at 80 μm with a focusing error *U_f_* = 10 μm/2 = 5 μm.

### 3.3. Results and Analysis of Subdivision with Focusing and CRM

After determining *i*_best_ = 9 and the optimal focal plane position, the F-P interference image shown in Figure 9 was obtained. Then, it was intercepted to be 1999 × 1999 pixels centred on the ring centre, which can reduce the calculation work while preserving the optimal ring. We conducted the subdivision using the CR and SCR methods and then calculated the standard deviation of the ring centre coordinates by CRM (see Section 2.3.1). The results are shown in Table 2. Different subdivision methods had the same line number of *N*′ = 50, which means that there were 404 ring peak coordinates on a single ring.

It can be seen from Table 2 that $s_{x_{0i}^{\prime \prime }}=s_{y_{0i}^{\prime \prime }}$ owing to the CRM characteristics. For the subdivision method, the pixel interval $w^{\prime }$ is $(1/\sqrt{2})w$ after using the CR method, and the pixel interval $w^{\prime }$ is reduced to w/2 after SCR. This is compared with the case of unsubdivision, and either the CR method or the SCR method can greatly improve the accuracy of the centre coordinates. In addition, the result from SCR is better.

### 3.4. Results and Analysis of MCAM and CRM with Focusing and SCR Subdivision

In Section 3.2 and Section 3.3, as it is well-known, the same line number *N*″ = 50, i.e., a total of 404 coordinates of ring peaks, were used for the calculation of the centre coordinates and their standard deviation. When adopting *N*″ = 6, 12, 25, 35, 50, 100, 200 to obtain the standard deviation of the centre coordinates from the fringe pattern after the SCR method, the CRM and the MCAM were both adopted for the calculation to better validate the influence of *N*″. The results are shown in Table 3 in units of $w^{\prime \prime }$. It can be seen that the standard deviation of the ring centre decreases with an increase in *N*″. When *N*″ = 50, the standard deviation of the centre of the two circle fitting methods can be maintained within $0.0060\times w^{\prime \prime }\approx 10\;\mathrm{nm}$, which basically meets the accuracy requirements of the microdisplacement. It can be clearly seen from Table 3 that when *N*″ is greater than 50, the decrease rate of the standard deviation slows down.

At the same time, consideration should be given that the larger *N*″ is, the longer the data processing time will be. In order to reduce the amount of data and speed up the operation speed, *N*″ = 50 is preferred. Finally, the centre coordinates ($x_{0i}^{\prime \prime }$ and $y_{0i}^{\prime \prime }$), with standard deviations ($S_{x_{0i}^{\prime \prime }}$ and $S_{y_{0i}}^{\prime \prime }$), and the radius $r_{i}$ with standard deviation $s_{r_{i}}$ were calculated under the CRM and MCAM methods, as shown in Table 4. From the results, the standard deviations of the centre coordinates are smaller in MCAM than in CRM, which means that MCAM is more suitable for the circle fitting to obtain the centre coordinates, and the centre coordinates and circle radii from the MCAM and CRM methods are different, but it does not matter whether the displacement measurement for the displacement is changed. In addition, the standard deviations of $x_{0i}^{\prime \prime }$ and $y_{0i}^{\prime \prime }$ calculated from MCAM are slightly different, which can indirectly reflect the *x*-direction and *y*-direction errors and benefit the analysis and adjustment of the experimental setup.

### 3.5. Experiments and Analysis of Microdisplacement Measurement

The microdisplacement measurement experiments were conducted using the SCR method and MCAM of circle fitting. After using the F-P etalon, imaging lens, and area-array detector, we can roughly obtain the measurement range of displacement, that is, the distance that the electric translation stage can move when the 9th ring can be found. Under current device conditions, the size of the area array is 17.4 × 13.0 mm, and the diameter of the 9th ring is about 1638$w^{\prime \prime }$ = 1638 × 2 × 3.35/2 = 5.5 mm. Therefore, theoretically, the measurement range can reach more than 5 mm.

However, owing to the existence of $\beta_{x}$ and $\beta_{y}$, which leads to the Abbe error, the interference imaging quality closer to the edge is theoretically worse. In the experiments, we performed two sets of displacement experiments. In the small-range microdisplacement experiment, we moved the electric translation stage from the initial position along the *x*-direction to 5, 10, 15, 20, 25, 30, and 35 μm, and the results in Table 5 were obtained. In the large-range displacement experiment, from a selected position to 1, 2, 3, 4, and 5 mm, the results are shown in Table 6. All results are the average values of three measurements at each position at an interval of 30 s. The displacement δ was calculated using Equation (2), and the calculation formula for the standard deviation of the displacement $S_{\delta }$ based on Equation (2) is as follows:

(18) $$S_{\delta }=\sqrt{{\left(\frac{y_{01}-y_{0}}{\delta }\right)}^{2}\times (S_{y_{01}}^{2}+S_{y_{0}}^{2})+{\left(\frac{x_{01}-x_{0}}{\delta }\right)}^{2}\times (S_{x_{01}}^{2}+S_{x_{0}}^{2})}$$

From Table 5, it is easily seen that the micro-range displacement can be measured from the discussed processing above with a small standard deviation of approximately 0.010 μm, and its excellent linearity has been previously verified [33]. If the uncertainty is extended to *k* = 2, then the extended uncertainty $U_{\delta }\approx 0.02\;\mu m$. Compared with the results in Table 6, for a large range of 5 mm, the extended uncertainty was approximately $U_{\delta }\approx 0.03\;\mu m$. This is completely reasonable for existing errors. However, the results obtained in this study provide good results.

## 4. Conclusions

This paper focused on a data processing method for determining the centre of the interference ring in an F-P multibeam interference imaging system. The nanometer-order centre coordinates were determined by selecting the optimal ring, precise focusing, virtual pixel subdivision, circle fitting, etc. Microdisplacement measurements using the experimental device were carried out and researched. The conclusions are as follows:

(1) Both the CR method and the SCR method of subdivision of pixels can improve the accuracy of the centre coordinate calculation of the concentric interference ring, and the SCR method performs better. The MCAM method of circle fitting is more suitable for calculating the circle centre coordinates than the CRM, and MCAM can achieve higher accuracy in calculating the circle centre coordinates.

(2) Under the current experimental conditions of light source, F-P etalon, imaging lens, area-array detector, etc., the standard experimental deviation of the centre of the circle is approximately $0.005\times w^{\prime \prime }\approx 0.009$ μm. Within the (0–5) mm measurement range, the extended uncertainty of the displacement measurement result is approximately $U_{\delta }\approx 0.03\;\mu m$ (*k* = 2). If we improve the environmental conditions of the laboratory and improve the component parameters and performance of the experimental equipment, then we fully believe that better results can be obtained. For example, when the average pixel interval w of the area array device might shrink to w/2, the standard experimental deviation of the circle centre 0.009 μm might be 0.0045 μm.

## Figures and Tables

**Table 1:** FWHM of peak position of 9th ring at different focusing positions.

Relative focusing distance (µm)	0	10	20	30	40	50	60	70	80
FWHM (*w*)	6.524	5.94	4.914	4.973	4.644	4.379	4.553	4.183	3.949
Relative focusing distance (µm)	90	100	110	120	130	140	150	160	170
FWHM (*w*)	4.046	4.117	4.7	4.907	5.452	5.13	6.41	7.096	6.099

**Table 2:** Two standard deviations of ring centre coordinates with or without subdivision (*i*_best_ = 9).

Parameters	With Unsubdivision	With CR Method	With SCR Method
Pixel interval	w	$w/\sqrt{2}$	w/2
$s_{y_{0i}^{\prime \prime }}$	0.0307*w*	0.0157*w*″ ≈ 0.0111*w*	0.0060*w*″ ≈ 0.0030*w*
$s_{x_{0i}^{\prime \prime }}$	0.0307*w*	0.0157*w*″ ≈ 0.0111*w*	0.0060*w*″ ≈ 0.0030*w*

**Table 3:** Standard deviations at different line numbers *N*″ (*i*_best_ = 9).

Method	Standard Deviation of the Centre	*N*″ = 6	*N*″ = 12	*N*″ = 25	*N*″ = 35	*N*″ = 50	*N*″ = 100	*N*″ = 200
CRM	$s_{x_{0i}^{\prime \prime }}$ ($w^{\prime \prime }$)	0.0147	0.0106	0.0085	0.0070	0.0060	0.0044	0.0032
	$s_{y_{0i}^{\prime \prime }}$ ($w^{\prime \prime }$)	0.0147	0.0106	0.0085	0.0070	0.0060	0.0044	0.0032
MCAM	$s_{x_{0i}^{\prime \prime }}$ ($w^{\prime \prime }$)	0.0120	0.0086	0.0069	0.0057	0.0047	0.0038	0.0028
	$s_{x_{0i}^{\prime \prime }}$ ($w^{\prime \prime }$)	0.0153	0.0102	0.0065	0.0050	0.0042	0.0029	0.0025

**Table 4:** Calculation results of ring information using two circle fitting methods (*i*_best_ = 9).

**Method**	$x_{0i}^{\prime \prime }(w^{\prime \prime })\;$	$y_{0i}^{\prime \prime }(w^{\prime \prime })\;$	$S_{x_{0i}^{\prime \prime }}(w^{\prime \prime })$	$S_{y_{0i}}^{\prime \prime }(w^{\prime \prime })$	$r_{i}(w^{\prime \prime })\;$	$s_{r_{i}}(w^{\prime \prime })$
CRM	2000.272	2000.352	0.0060	0.0060	1638.004	0.0042
MCAM	2000.270	2000.348	0.0047	0.0042	1637.882	0.0033

**Table 5:** Small range displacement experiment results at 35 μm (*i*_best_ = 9).

Relative Moving Distance	$x_{0i}^{\prime \prime }(w^{\prime \prime })$	$S_{x_{0i}^{\prime \prime }}(w^{\prime \prime })$	$y_{0i}^{\prime \prime }(w^{\prime \prime })$	$S_{y_{0i}}^{\prime \prime }(w^{\prime \prime })$	δ (μm)	$S_{\delta }\;(\mu m)$
0 μm	2000.270	0.0047	2000.348	0.0042	0	/
5 μm	2002.632	0.0042	1998.358	0.0040	5.17	0.010
10 μm	2004.620	0.0031	1996.104	0.0031	10.18	0.009
15 μm	2006.854	0.0044	1993.992	0.0039	15.33	0.010
20 μm	2008.754	0.0036	1991.550	0.0036	20.47	0.010
25 μm	2010.890	0.0042	1989.358	0.0035	25.60	0.010
30 μm	2012.972	0.0041	1987.152	0.0029	30.68	0.010
35 μm	2015.178	0.0030	1985.016	0.0036	35.82	0.010

**Table 6:** Large range displacement experiment result at 5 mm (*i*_best_ = 9).

Relative Moving Distance	$x_{0i}^{\prime \prime }(w^{\prime \prime })$	$S_{x_{0i}^{\prime \prime }}(w^{\prime \prime })$	$y_{0i}^{\prime \prime }(w^{\prime \prime })$	$S_{y_{0i}}^{\prime \prime }(w^{\prime \prime })$	δ (μm)	$S_{\delta }\;(\mu m)$
0 mm	3546.566	0.0047	1556.957	0.0065	0	/
1 mm	2941.740	0.0060	1540.841	0.0041	1013.44	0.013
2 mm	2336.960	0.0054	1524.909	0.0035	2026.80	0.012
3 mm	1731.976	0.0063	1509.061	0.0035	3040.50	0.013
4 mm	1126.774	0.0074	1492.751	0.0050	4054.58	0.015
5 mm	521.766	0.0055	1476.867	0.0067	5068.32	0.012

## Data Availability

The data presented in this study are available in article.
